# Who benefits from self-management support? Results from a randomized controlled trial

**DOI:** 10.1016/j.heliyon.2023.e17752

**Published:** 2023-06-28

**Authors:** Kirstine Skov Benthien, Camilla Palmhøj Nielsen, Knud Rasmussen, Kristian Kidholm, Mette Grønkjær, Ulla Toft

**Affiliations:** a Palliative Care Unit, Copenhagen University Hospital, Hvidovre, Denmark; b Center for Clinical Research and Prevention, Copenhagen University Hospital - Frederiksberg, Denmark; c DEFACTUM - Social & Health Services and Labour Market, Aarhus, Denmark; d Department of Public Health, Aarhus University, Denmark; e Data and Development Support, Region Zealand, Sorø, Denmark; f Centre for Innovative Medical Technology, Odense University Hospital, Denmark; g Clinical Nursing Research Unit, Aalborg University Hospital, Denmark; h Department of Clinical Medicine, Aalborg University, Denmark

**Keywords:** Tele-health, Self-management support, Randomized controlled trial

## Abstract

**Background:**

Self-management support models adapted to accommodate the needs of each patient are complex interventions that should be evaluated for intervention mechanisms. In a national randomized controlled trial (RCT), we evaluated the efficacy of telephone-based self-management support that demonstrated improved health-related quality of life (HRQoL), no reduction in hospital admissions, and an unexpected increase in primary healthcare services.

**Objective:**

The objective of this study is to identify RCT impact mechanisms and explore which participants could benefit the most from the PaHS intervention.

**Methods:**

This study evaluates intervention mechanisms through interaction analyses of predefined intervention moderators (sex, age, education, chronic disease, risk of hospital admissions, and coping) and post-hoc intervention mediators (contacts in primary care and anxiety medication). The one co-primary outcome HRQoL was assessed with SF26v2 and analyzed with generalized linear mixed models and the other co-primary hospital admissions was analyzed with poisson regression.

**Results:**

PaHS interacted with diabetes, multimorbidity, coping, and anxiety medication on the outcome hospital admissions. PaHS led to a significant reduction in hospital admissions in participants with diabetes or multimorbidity and an increase in hospital admissions in participants with higher baseline coping and participants using anxiety medication. The interaction analyses revealed significant intervention mediation in the outcome HRQoL by sex and diabetes.

**Conclusions:**

Participants with diabetes, multimorbidity, and women could benefit the most from telephone-based self-management support, but the intervention involves the risk of over-treatment.

## 1 Introduction

Self-management support (SMS) interventions that are person-centered and adapted to accommodate the needs of each patient meet several characteristics of complex interventions: they require skills and behaviors by patient as well as healthcare providers, contain several components, and require flexibility [1]. Complex interventions are evaluated not only for efficacy or effectiveness, but the ‘how’ and ‘why’ should be explored when the intervention is embedded in a system with unanticipated, emergent and feedback mechanisms [1]. We previously evaluated a generic telephone-based self-management support intervention (Proactive Health Support (PaHS)) in a national randomized controlled trial (RCT) (ClinicalTrials.gov, NCT03628469) [2]. PaHS improved health-related quality of life (HRQoL), but could not reduce hospital admissions as intended [3]. Interaction and subgroup analyses of the most prevalent chronic diseases revealed no interaction between heart disease, pulmonary disease, or connective tissue disease but a significant interaction between diabetes and intervention effect on HRQoL and hospital admissions [3]. Secondary RCT-endpoints revealed an unanticipated increased use of anxiety medication and primary healthcare services, particularly psychologists [3]. The aim of this study was to identify PaHS RCT impact mechanisms and thereby explore which participants could benefit the most from a generic telephone-based self-management support intervention.

## 2 Methods

This study of RCT impact mechanisms includes analyses of moderators and mediators. Moderators are baseline characteristics that interact with the intervention effect, whereas mediators are active organisms that intervene between intervention and outcome [4]. This study includes analyses of 1. baseline participant characteristics predefined in the protocol [2] as candidates of intervention effect moderators and 2. potential intervention effect mediators identified post-hoc in the trial evaluation as secondary endpoints affected by the intervention.

### 2.1 Main RCT methods

The RCT is described in detail in the protocol paper and main evaluation paper [2,3]. Briefly, the study is a national RCT of PaHS versus usual universal tax-funded healthcare. Participants were elderly, had chronic diseases, or frequent emergency contacts and an increased risk of emergency hospital admissions identified with a prediction algorithm [5]. The intervention was implemented 9 months prior to RCT [6] and began with a physical start-up session to ensure a trusting relationship between participant and provider [7] followed by telephone sessions of self-management support [8] in which one or more action plans were decided. The intervention group received a median of 9 follow-up sessions.

### 2.2 Hypothesized intervention effect moderators and mediators

Predefined baseline variables tested for intervention moderation were number of diagnoses, most prevalent chronic diagnoses, age, sex, level of education, marital status, and predicted risk of hospital admission. These baseline variables were included in all analyses as covariates. Furthermore, baseline coping assessed with two scales of the Health Education Impact Questionnaire [9] were included: Positive and Active Engagement in Life and Skill (PAEL) and Skill and Technique Acquisition (STA). For both scales, higher is better. Secondary RCT-outcomes with statistically significant intervention effect were tested post-hoc as potential mediators: Services by general practitioner (GP), other medical specialists, psychologists, and use of anxiety medication. Furthermore, number of telephone counseling sessions was predefined in the protocol article and is by temporal sequence a mediator.

### 2.3 Outcomes

The co-primary outcomes of the trial were hospital admissions and the Mental Health Composite Score of SF36 v2 [10] at six months follow-up. For the Mental Health Composite Score, higher is better.

### 2.4 Analyses

The analyses were based on the intention-to-treat principle modified by excluding participants who withdrew consent. Mediation and moderation analyses were selected for consistency with the main RCT-analyses. The analyses of HRQoL-outcomes were generalized linear mixed models with autocorrelation and unstructured covariance. These models assume that missing data is related to covariates or the previously observed values of same items, which infers multiple imputation of missing follow-ups. Intervention moderation and mediation was modeled with a three-way interaction analysis of time x group x ‘moderator/mediator’. The analyses of hospital admissions were poisson regression and intervention moderation was modeled with interaction of group x ‘moderator/mediator’. The algorithm for predicting risk of hospitals admissions had regional variation due to differing populations and access to statistical software and this moderator was therefore analyzed regionally as well as nationally. Since number of telephone counseling sessions only applied for the intervention group, this group was divided into persons with up to 5 and persons with more than 5 telephone sessions for comparison with the control group.

### 2.5 Ethics

This study was approved by The Zealand Regional Committee on Health Research Ethics (SJ-677). All participants provided written, informed consent.

## 3 Results

The interaction analyses revealed significant intervention mediation in the outcome hospital admissions by diabetes, multimorbidity, and STA and intervention moderation by anxiety medication. PaHS led to a significant reduction in hospital admissions in participants with diabetes or multimorbidity and an increase in hospital admissions in participants with higher baseline STA. PaHS led to a significant increase in hospital admissions in participants who filled prescriptions on anxiety medication. The interaction analyses revealed significant intervention mediation in the outcome HRQoL by sex and diabetes. While the intervention improved HRQoL for both sexes, the effect was larger for women and participants with diabetes. Interaction analyses are presented in Table 1.

**Table 1:** Proactive Health Support moderators and mediators.

	Hospital admissions^a^				Mental Health Composite Score^b^			
	Estimate	95% Confidence intervals	SE	*P*-value	Estimate	95% Confidence intervals	SE	*P*-value
Sex (Female)	0.0479	(-0.1298, 0.2256)	0.0907	0.5975	1.2469	(0.08275, 2.4111)	0.5939	**0.0358**
Age (70+)	0.1375	(-0.0394, 0.3145)	0.0903	0.1277	−0.5470	(-1.7174, 0.6234)	0.5971	0.3596
Marital status (Married)	−0.1235	(-0.3014, 0.0544)	0.0908	0.1735	0.3890	(-0.7932, 1.5713)	0.6032	0.5189
Level of education								
Primary and lower secondary	Reference				Reference			
Post-secondary (Vocational) and upper secondary	0.0349	(-0.1666, 0.2364)	0.1028	0.7344	0.2882	(-1.1028, 1.6791)	0.7096	0.6847
Short tertiary, bachelor's, Master's level or above	−0.0544	(-0.2938, 0.1850)	0.1221	0.6559	0.1962	(-1.3688, 1.7613)	0.7984	0.8059
Chronic diseases								
Diabetes	−0.2525	(-0.4431, −0.0619)	0.0972	**0.0094**	1.3189	(0.03485, 2.6029)	0.6551	**0.0441**
Heart diseases	−0.0913	(-0.2685, 0.0858)	0.0904	0.3124	−0.9887	(-2.1717, 0.1943)	0.6035	0.1014
Connective Tissue diseases	0.0220	(-0.1546, 0.1987)	0.0901	0.8071	0.9891	(-0.1815, 2.1597)	0.5972	0.0977
Pulmonary diseases	0.0443	(-0.1333, 0.2220)	0.0906	0.6247	0.2589	(-0.9330, 1.4509)	0.6081	0.6703
Multimorbidity	−0.3368	(-0.5624, −0.1113)	0.1151	**0.0034**	0.8676	(-0.4051, 2.1403)	0.6496	0.1815
Health Education Impact Questionnaire								
Positive and Active Engagement in Life (3+)	0.0238	(-0.1578, 0.2055)	0.0927	0.7969	−0.0048	(-1.2764, 1.2668)	0.6487	0.9941
Skill and Technique Acquisition (3+)	0.1842	(0.0072, 0.3611)	0.0903	**0.0414**	−1.2986	(-3.0589, 0.4618)	0.8981	0.1482
Predicted risk of hospital admissions								
<25	Reference				Reference			
25–49	−0.2335	(-0.4852, 0.0183)	0.1285	0.0692	0.4620	(-1.3017, 2.2258)	0.8998	0.6076
50+	−0.1156	(-0.3337, 0.1026)	0.1113	0.2290	−0.5054	(-1.8782, 0.8673)	0.7003	0.4705
Contact to psychologist	−0.2613	(-0.6996, 0.1771)	0.2237	0.2427	0.8020	(-2.4053, 4.0093)	1.6362	0.6240
GP services (30+)	−0.0336	(-0.2109, 0.1436)	0.0904	0.7101	−0.4691	(-1.6636, 0.7253)	0.6094	0.4414
Other medical specialist services (3+)	0.0929	(-0.0862, 0.2719)	0.0913	0.3094	−0.1461	(-1.3145, 1.0222)	0.5961	0.8063
Anxiety medication	0.3295	(0.1381, 0.5209)	0.0977	**0.0007**	−0.3567	(-1.8614, 1.1479)	0.7676	0.6421

^a^Generalized linear models with poisson distribution of interaction between group x ‘moderator/mediator’, ^b^Mixed models of three-way interaction analysis of group x time x ‘moderator/mediator’. All analyses are at six months follow-up and adjusted for sex, age, education, marital status, region, number of chronic diseases (diabetes, heart, connective tissue, pulmonary, renal, neurologic, gastrointestinal, and cancer), and multimorbidity.

Subgroup analyses of participants with diabetes were presented in the primary article [3]. Regional subgroup analyses of the predicted risk of hospital admissions revealed negative coefficients indicating reduced hospital admissions in the subgroup with a predicted risk of hospital admission of 25–49% in four of five regions, which was highly statistically significant in two regions (Zealand and Southern Denmark) (Data not shown). Number of hospital admissions and HRQoL was not associated with number of telephone counseling sessions (data not shown).

## 4 Discussion

These analyses of intervention mechanisms have revealed subgroups that may benefit from the generic telephone-based self-management support intervention, PaHS, including participants with diabetes, multimorbidity, and women. Participants with a high baseline STA may experience an adverse reaction where the intervention amplifies health seeking behavior leading to increased hospital admissions. Participants who fill anxiety prescription during PaHS are at risk of increased number of hospital admissions due to the intervention even controlling for anxiety medication alone. The main article demonstrated an increased use of psychologists, anxiety medication, and other medical specialists in the intervention group [3]. The present study has clarified that this increased use was not associated with improved intervention benefit. This indicates the risk of over-treatment as a consequence of the PaHS intervention. The analyses in this study were secondary and exploratory and contain the risk of Type-II errors. Furthermore, the analyses were limited to the primary outcomes and the secondary outcomes may be hold further information about intervention mechanisms. The results may be used for hypothesis generation and future intervention development. Further trials are needed to investigate the potential for effectiveness and cost-saving in the identified benefitting subgroups. This includes studies of cost-effectiveness since PaHS involves substantial personnel resources.

## Author contribution statement

Kirstine Benthien: Conceived and designed the experiments; Performed the experiments; Analyzed and interpreted the data; Contributed reagents, materials, analysis tools or data; Wrote the paper.

Camilla Palmhøj Nielsen, MSc, PhD; Knud Rasmussen, MD, DMSc; Kristian Kidholm, Professor; Mette Grønkjær, Professor; Ulla Toft, MSc, PhD: Conceived and designed the experiments; Performed the experiments; Contributed reagents, materials, analysis tools or data; Wrote the paper.

## Funding statement

The PaHS RCT is funded by an unrestricted grant from the Danish Ministry of Health. The sponsor has no role in trial management, analysis, and interpretation of data.

## Data availability statement

The data that has been used is confidential.

## Declaration of competing interest

The authors declare that they have no known competing financial interests or personal relationships that could have appeared to influence the work reported in this paper.
